# Efficacy analysis of a double-Schanz screw external fixator combined with anti-rotating Kirschner wire in the treatment of proximal humerus fractures in skeletally immature patients

**DOI:** 10.1186/s13018-022-03434-5

**Published:** 2022-12-16

**Authors:** Qian Wang, Yu Wang, Huai Zhao, Qingzhu Kong, Jingxin Zhao, Yu jin

**Affiliations:** 1 Trauma Department of Orthopedics, Affiliated Hospital of Chengde Medical College, 36 Nanyingzi Street, Shuangqiao District, Chengde, 067000 Hebei People’s Republic of China; 2 Department of General Surgery, Shengjing Hospital Affiliated China Medical University, Shenyang, 110004 Liaoning People’s Republic of China

**Keywords:** Proximal humerus fracture, Children, Adolescent, External fixator

## Abstract

**Objectives:**

The objective of this study is to evaluate the efficacy of a double-Schanz screw external fixator combined with anti-rotating Kirschner wire in the treatment of displaced Salter–Harris type II proximal humerus fractures in skeletally immature patients.

**Methods:**

A retrospective analysis was performed on 22 cases of displaced Salter–Harris type II proximal humerus fractures in skeletally immature patients who were treated with a double-Schanz screw external fixator combined with anti-rotating Kirschner wire. Patients included were the Neer–Horowitz (N–H) type 2, 3, and 4 of fracture. The basic information of the patients was recorded, fracture healing and shoulder range of motion were assessed at the last follow-up visit. The disabilities of the arm, hand (DASH) score and Constant—Murley score of the shoulder were performed to observe the occurrence of complications.

**Results:**

The mean age at the time of surgery was 12.41 years, and all patients completed a median follow-up of 18.18 months. There were two cases of N–H type 2, 12 cases of N–H type 3, and eight cases of N–H type 4 among the patients. At the last follow-up, all patients were able to achieve pain-free shoulder movement. There was no significant difference in shoulder function between the injured side and the uninjured side. The DASH score mean was 2.43 (95% CI 1.44–3.52). The constant score mean was 98.55 (95% CI 97.73–99.27). All patients returned to their pre-injury daily life and physical activities, and there was no significant difference in bilateral limb length at the last follow-up (*p* < 0.05). The most common complication of double-Schanz screw external fixator combined with anti-rotating Kirschner wire surgery was pin tract infection, which occurred in 5 cases (22.7%). There were no complications such as deep infections, vascular and nerve damage, failure of fixation, secondary fracture displacement, non-union of fracture, osteonecrosis of the humerus, joint stiffness, rotator cuff weakness and limb deformity.

**Conclusion:**

The double-Schanz screw external fixator combined with anti-rotating Kirschner wire is a safe and effective treatment for displaced Salter–Harris type II proximal humerus fractures in skeletally immature patients over the age of 10 years.

## Introduction

Proximal humeral fractures are not as common as supracondylar or distal radius fractures in children [1], but they are also a major type of pediatric fracture. Proximal humerus fractures account for approximately 2–6% of all fractures in children and typically occur in children and adolescents between the ages of 10–14 [1–3]. The most commonly used classifications for proximal humeral fractures in children are the Neer–Horowitz (N–H) classification and the Salter–Harris classification. The N–H classification divides proximal humeral fractures into 4 types: type 1: fracture displacement < 5 mm; type 2: fracture displacement up to 1/3 of the width of the humeral shaft; type 3: fracture displacement up to 2/3 of the width of the humeral shaft; type 4: fracture displacement > 2/3 of the width of the humeral axis.

Salter–Harris type II fractures are primarily fracture type in older children and adolescents [4, 5], accounting for 4–7% of pediatric epiphyseal fractures [6]. The shoulder capsule insertion extends vertically downwards along the lateral edge of the epiphyseal plate to the medial side of the metaphysis. This anatomical structure explains the high proportion of Salter–Harris type II epiphyseal separation in proximal humeral fractures [7].

Fractures of the proximal humerus in children under 10 years of age are usually treated conservatively (e.g., casts, slings, etc.) because of the strong molding capacity of the proximal humerus at this age. The proximal humerus has great growth and plasticity potential, which accounts for 80 percent of the length of the humerus. In addition, the shoulder joint has a large range of motion, which can compensate for some degree of skeletal deformity [8].

For proximal humeral fractures in adolescents, which patients need surgery and which surgical method is best remains controversial. Because the adolescent bone is closer to maturity and its plasticity is significantly reduced after fracture, which increases the risk of poor prognosis, numerous current studies support aggressive surgical treatment for displaced proximal humerus in adolescents [4, 9] to achieve better fracture healing and functional outcomes. At present, the surgical fixation methods used include Kirschner wire, elastic stable intramedullary nails, cannulated screw and locking compression plate, etc. All kinds of fixation methods have their advantages and disadvantages, but no unified consensus has been formed, and the "gold standard" for surgical treatment has not been established. As an attempt at technical innovation, in recent years we have used a double-Schanz screws external fixator in combination with anti-rotating Kirschner wire for the treatment of proximal humeral fractures in skeletally immature patients over 10 years old. The purpose of this study is to analyze the efficacy of the double-Schanz screw external fixator combined with an anti-rotating Kirschner wire for the treatment of Salter–Harris II proximal humeral fractures in older children and adolescents.

## Methods

### Patients

The clinical data of 22 skeletally immature patients aged > 10 years with Salter–Harris II proximal humerus fracture who were treated at the Affiliated Hospital of Chengde Medical College from June 2019 to April 2022 were analyzed retrospectively. Patients included were the Neer–Horowitz (N–H) type 2, 3, and 4 of fracture. Children with open fractures or fractures from other bone or organ injuries were excluded. All patients were treated with a double-Schanz screw external factor in combination with an anti-rotating Kirschner wire. The demographic and clinical characteristics of the patients are summarized in Table 1. Formal consent was obtained from a parent or guardian. The study was reviewed and approved by the institutional ethics committee.

**Table 1:** Demographic and clinical date of patients

Number	Sex	Age (years)	Side	Height (cm)	Weight (kg)	BMI	Neer–Horowitz type	Operation time (min)	Anesthesia time (min)	Open reduction	Hospitalization time (d)	Follow up time (m)
1	M	11	R	160	47	18.36	2	40	60	Y	2	6
2	F	11	L	155	92	38.29	4	50	110	Y	6	16
3	M	11	L	145	45	21.40	3	63	98	N	3	17
4	M	12	L	150	60	26.67	3	102	130	N	4	20
5	M	12	L	170	80	27.68	3	120	160	Y	5	17
6	M	15	R	170	56	19.38	4	35	70	N	5	16
7	M	13	R	160	50	19.53	3	34	70	N	4	18
8	M	15	L	176	75	24.21	4	94	155	N	11	18
9	M	11	L	152	41	17.75	3	50	100	N	3	17
10	F	14	L	165	47	17.26	4	40	59	N	6	18
11	M	16	L	180	64	19.75	4	70	90	N	5	19
12	M	12	L	163	61	22.96	3	35	68	N	6	20
13	F	14	L	162	63	24.01	3	66	80	N	7	19
14	M	13	L	160	110	42.97	4	55	105	Y	4	19
15	M	13	L	155	58	24.14	3	65	95	N	6	20
16	M	12	R	160	50	19.53	3	50	99	N	6	20
17	M	12	R	145	45	21.40	3	55	110	N	3	20
18	M	12	L	162	72	27.43	4	103	150	Y	11	20
19	F	11	L	145	40	19.02	3	80	113	N	5	20
20	M	13	R	160	50	19.53	4	69	120	Y	3	20
21	M	10	R	150	40	17.78	3	45	105	N	4	20
22	M	10	L	160	47	18.36	2	40	60	N	2	20
Mean		12.41		159.32	58.77	23.06		61.86	100.32		5.45	18.18
SD		1.62		9.45	9.45	17.89		24.54	30.14		2.38	3.06

*F* female, *M* male, *R* right, *L* left, *Y* yes, *N* no, *m* month

### Surgical technique

Patients were placed in a supine position on an operating bed after general anesthesia. ​The elbow joint was flexed, the upper arm was drawn longitudinally in an internal rotational position, and the humerus shaft was simultaneously pushed back. Fractures were usually reduced by abduction. After a satisfactory reduction, the reduction was confirmed by C-arm fluoroscopy.

Subsequently, we inserted a 4.0 mm cancellous bone Schanz screw in the humeral head, which was drilled with a drill bit before insertion and positioned with a 2.5 mm Kirschner wire before insertion, ensuring that the Kirschner wire was positioned above the proximal epiphysis plate of the humerus, did not penetrate the articular cartilage, and served as a substitute for the drill bit. After the Kirschner wire had been inserted properly, we inserted the teeth of the sleeve along the end of the Kirschner wire and gently tapped it tightly. The Kirschner wire was then removed to ensure that the position of the sleeve remains the same and a Schanz screw was inserted. C-arm fluoroscopy was used to ensure the proper position and depth of the Schanz screw. If the closed reduction was difficult (tried 3 attempts), open reduction was performed. A small incision of 2–3 cm was made in the epiphysis of the humeral diaphysis. Under the protection of the sleeve, a 4.0 mm diameter self-drilling Schanz screw was directly inserted into the distal part of the fracture using an electric drill. The external fixator was held in place by connecting rods after a fluoroscopic fracture reduction and satisfactory pinning position. Subsequently, one or two 1.8–2.0 mm anti-rotating Kirschner wires were inserted to secure the fracture, and the ends of the Kirschner wire were bent and clipped together with the Schanz screw on the external fixator frame to increase the fixation strength. After surgery, a simple sling was used to immobilize the shoulder joint, and moderate activity of the shoulder joint can be resumed after the pain has subsided.

### Postoperative follow-up and functional evaluation

Patients were reviewed by outpatient clinics in the second week, fourth week, second month and third month after surgery and then followed up irregularly. Meanwhile, instant reviews were conducted using WeChat. During the follow-up, the incidence of complications was observed and the healing of the fracture was observed by X-ray or CT of the shoulder joint. After the fracture healed, the fixation was removed. At the last follow-up, the shoulder function was comprehensively evaluated, bilateral upper arm length was measured, and the DASH score and Constant score of the shoulder were performed.

### Statistical analysis

The data were analyzed by SPSS 20.0 software. Descriptive statistics, including the calculation of the mean and standard deviation or 95% confidence interval, were performed for each examined variable. The postoperative upper limb length on the injured side was compared with the normal upper limb length using the independent samples *t*-test, and *P* < 0.05 was considered statistically significant.

## Result

A total of 22 skeletally immature patients with Salter–Harris type II proximal humeral fractures were included in this study, including 18 males (81.82%) and 4 females (18.18%). The mean age at surgery was 12.41 years, left side: right side = 15:7. There were two cases of N–H type 2, 12 cases of N–H type 3, and eight cases of N–H type 4 among the patients. All patients were followed up for an average of 18.18 months. The basic information about the patients is given in Table 1.

All patients were treated with a double-Schanz screw external fixator in combination with anti-rotating Kirschner wire, including 16 cases of closed reduction and 6 cases of open reduction. The barrier to closed reduction was the periosteum embedding. The average operation time was 61.86 min (34 min-120 min).

All fractures healed smoothly, and all patients were able to achieve pain-free shoulder joint movement at the final follow-up. In the meantime, the difference in shoulder joint movement between the injured and uninjured sides was compared. The DASH score mean was 2.43 (95% CI 1.44–3.52). The constant score mean was 98.55 (95% CI 97.73–99.27). All patients returned to their pre-injury daily life and physical activity. The range of motion of the joint and the length of the upper arm are shown in Table 2. There is no significant difference in bilateral limb length at the last follow-up (*p* < 0.05, Table 3).

**Table 2:** Range of motion of the affected shoulder joint at follow-up and scores

Number	Adduction (°)	Abduction (°)	Flexion (°)	Extension (°)	Internal rotation (°)	External rotation (°)	Horizontal anterior flexion (°)	Horizontal posterior extension (°)	Uninjured upper arm length (cm)	Injured upper arm length (cm)	DASH score	Constant score
1	45	90	177	48	55	45	130	45	31.6	31.5	6.67	95
2	40	85	172	50	50	40	125	40	28	27.8	0	100
3	40	85	170	40	50	45	130	45	25.5	25.6	1.67	100
4	45	85	172	48	55	40	125	50	29.5	29.2	5	95
5	50	85	180	50	60	45	130	45	34.8	34.6	0	100
6	50	95	180	55	60	40	135	50	33.5	33.8	6.67	96
7	45	95	175	50	55	43	130	50	32	32.1	2.5	100
8	50	90	180	55	55	45	125	45	36.1	35.6	4.17	98
9	45	95	178	45	58	40	130	48	25.8	25.5	0	100
10	45	90	175	45	50	45	130	48	30.5	30.2	6.67	96
11	50	85	178	50	55	40	125	48	34.2	34.2	0	100
12	45	90	178	48	55	40	135	48	31.5	31.6	3.33	98
13	50	90	178	50	60	40	130	48	31.4	31.2	1.67	100
14	45	85	175	50	50	43	130	45	33.2	32.8	0	100
15	45	90	179	50	58	40	130	50	31.8	31.4	3.33	98
16	40	85	170	48	52	40	125	45	32.3	32.0	0.83	100
17	45	95	175	50	55	40	125	50	25.6	25.1	0	100
18	45	95	175	48	60	43	130	50	32.2	31.6	4.17	98
19	45	85	178	49	60	43	135	45	25.5	25.3	0	100
20	50	90	180	55	60	45	135	50	30.8	30.5	5	96
21	45	90	178	50	60	43	130	50	29.5	29.1	1.67	98
22	50	95	178	50	58	45	135	48	30.2	29.8	0	100

The range of motion is the shoulder joint on the injured side

The smaller the DASH score, the better, with a minimum value of zero

The larger the constant score, the better, with a maximum of 100

**Table 3:** Comparison of clinical scores and limb lengths

	Mean	95% CI	Range	SD	*P*
DASH score	2.43	1.44–3.52	0–6.67		
Constant score	98.55	97.73–99.27	95–100		
Uninjured upper arm length (cm)	30.70	29.47–31.96	25.5–36.1	3.06	0.941
Injured upper arm length (cm)	30.48	29.17–31.83	25.1–35.6	3.08	

A *p* value < 0.05 was statistically significant

Complications: The most common complication of double-Schanz screw external fixator combined with anti-rotating Kirschner wire surgery was pin tract infection, which occurred in 5 cases (22.7%), including 3 cases in the pin tract of Schanz screw, and 2 cases with both Schanz screw and anti-rotating Kirschner wire. The infection recovered after a local dressing change and removal of the fixator. There were no complications such as deep infections, vascular and nerve damage, failure of internal fixation, secondary fracture displacement, non-union of fracture, osteonecrosis of the humerus, joint stiffness, rotator cuff weakness and limb deformity.

## Discussion

The management of severely displaced fractures in children over the age of 10 is controversial. Closure of proximal epiphyseal plate occurs in girls between the ages of 14 and 17 and boys between the ages of 16 and 18. Adolescents with severely displaced proximal humerus may benefit more from surgery because of their limited growth and remodeling potential [10].

There is no consensus in the literature regarding the indications for surgical treatment of proximal humerus fractures in older children and adolescents. Although some authors recommend surgical treatment for N–H type 3 and 4 fractures 10 years and older with > ​ 20 to 30 degrees of angulation, others recommend surgical treatment for patients 12 years and older with > 45 degrees of angulation [11]. Chaus et al. compared conservative and surgical treatment of proximal humerus fractures in children with N–H type 3 and 4 and found that the rate of unsatisfactory treatment outcomes increased significantly in patients > 12 years of age. For every 1-year increase in age at the time of injury, the likelihood of unsatisfactory treatment results increased by 3.81 times [6]. Hohloch et al. [12] considered that surgical treatment of proximal humeral fractures in children < 10 may be over-treated. Conservative management of proximal humeral fractures in patients 10 to 13 years of age was likely to result in limb shortening and residual deformity. Conservative management is not recommended for patients older than 13 years with displaced proximal humeral fractures. Other researchers recommend treatment depending on age and the degree of displacement of the fracture. Surgery was recommended for patients 10 to 13 years of age with fracture displacement greater than 50% and/or angulation greater than 40°, as well as for patients older than 13 years of age with fracture displacement greater than 30% and/or angulation greater than 20°. Treatment of proximal humeral fractures, especially in children and adolescents aged 10–13 years, should be individualized [7]. There are even studies that recommend surgery for all patients older than 12 years with a proximal humeral fracture [13].

There are also many surgical methods for proximal humeral fractures. This depends on several factors, including patient age, fracture type, bone quality, and surgeon preference (pediatric orthopedic surgeons may prefer to use elastic stable intramedullary nails [14]).

Kirschner wire fixation is the most common surgical fixation method for proximal humeral fractures in children [15]. Other researchers have used a single cannulated screw to fix Salter–Harris type II proximal humeral fractures in adolescents because the strength of the cannulated screw is greater than that of the Kirschner wire [16]. Meanwhile, elastic stable intramedullary nails (ESINs) have been used to treat severely displaced proximal humerus fractures with satisfactory results. The researchers then compared the outcomes of different surgical procedures. Kraus et al. [17] compared the therapeutic effects of Kirschner wire and ESINs in the surgical treatment of N–H type 3 and 4 proximal humeral fractures in adolescents, and the results showed that the operation time of elastic intramedullary nail was shorter than that of Kirschner wire, but the hospital stay and implant removal time were longer, and there was no significant difference in shoulder joint function scores between the two at follow-up. In another study with a follow-up of 9 months, Hutchinson et al. [14] also applied the two surgical methods in the treatment of N–H type 4 proximal humeral fractures in the adolescent with fracture angulation ≥ 40 degrees and found that the incidence of postoperative complications of ESINs was lower than that of Kirschner wire. However, the ESINs operation increased in time and blood loss, and a second operation was required to remove the internal fixation.

**Fig. 1:**
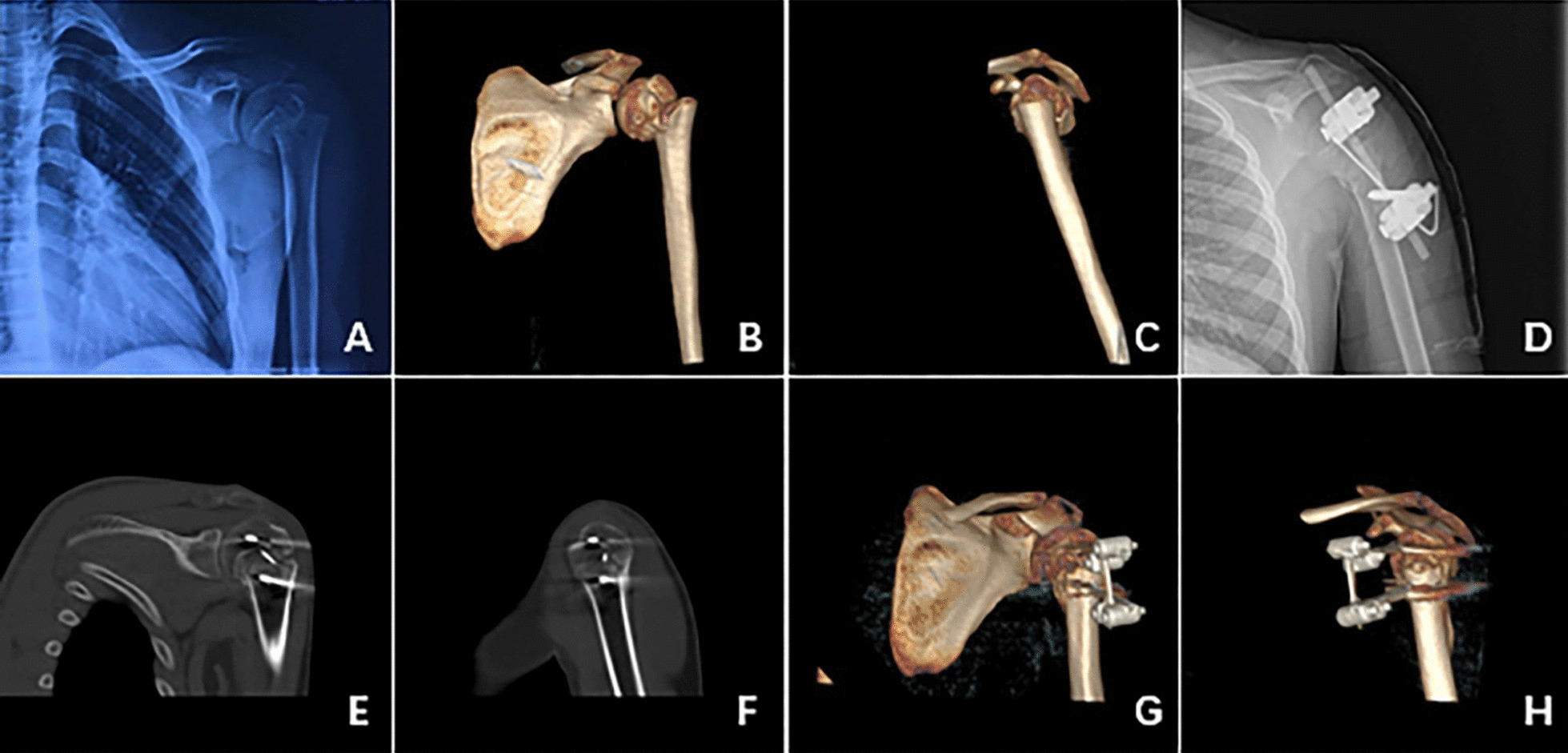
**A** Preoperative X-ray of a 15-year-old male patient with Salter–Harris II displaced proximal humeral fracture. **B** and **C** Preoperative 3D-CT of proximal humerus fracture. **D** Postoperative X-ray of proximal humerus fracture with the double-Schanz screw external fixator combined with anti-rotating Kirschner wire. **E** and **F** Postoperative 2D-CT of proximal humeral fracture (6 weeks after surgery). **G** and **H** Postoperative 3D-CT of proximal humerus fracture (6 weeks after surgery)

**Fig. 2:**
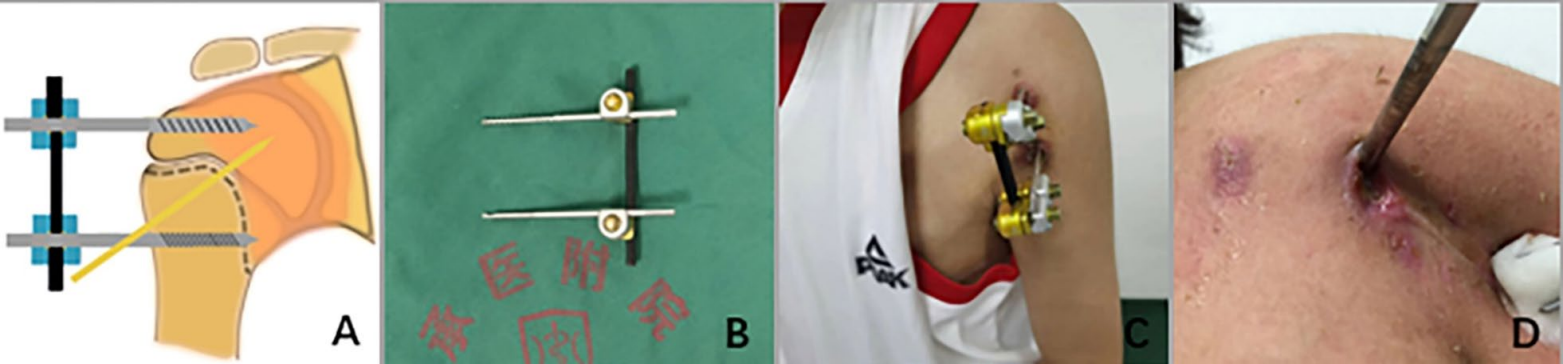
**A** Schematic diagram of external fixator and anti-rotating Kirschner wire for proximal humerus fracture. **B** Configuration of the external fixator (anti-rotating Kirschner wire was not included). **C** Postoperative appearance of external fixator combined with anti-rotating Kirschner wire. **D** A case of pin tract infection occurred after surgery

There are few studies on external fixators for proximal humeral fractures. Blonna et al. [18] performed fracture reduction and external fixator surgery on 188 adult patients with proximal humerus fractures by inserting more than 2.5 mm Kirschner wires into the proximal and distal ends of the fractures and then connecting and fixing the end of the Kirschner wires with a connecting rod. Although some complications of pin-track infection occurred (8.1%), the overall treatment effect was satisfactory. Regarding the surgical treatment of proximal humerus fractures in children, Lollino N et al. described two cases of external fixation for the treatment of Salter–Harris type II proximal humeral fractures in adolescents in 2013, After the fracture reduction, they secured the broken ends with four 2.5 mm Kirschner wires. The end of the Kirschner wire is then secured with a connecting rod to form an external fixator. They concluded that this form of external fixator provided better stability, but the disadvantage was that the number of cases included was too small [19]. Then Li et al. [20] compared the effects of external fixator and Kirschner wire in the treatment of proximal humerus fractures in adolescents. The external fixation architecture they used was two Schanz screws at each end of the fracture. The two Schanz screws at the proximal end of the fracture were placed at different angles in the same horizontal plane, and the other two Schanz screws at the distal end of the fracture are fixed in the distal end of the fracture at the appropriate spacing. The four Schanz screws were then connected to form an external fixator. Their results compared with those of the external fixator group, the operative time and the number of intraoperative fluoroscopies were significantly lower than those of the Kirschner wire group, and the rate of open reduction was lower in the external fixator group due to the joystick effect of the Schanz screws. They considered the external fixator to be superior to the Kirschner wire. The configuration of the external fixator we used was inspired by the external fixator used by Professor Slongo in the supracondylar fracture of the humerus [21]. This configuration is easy to operate and allows early movement of the affected limb after surgery. At the same time, there is no need for a second operation to remove the fixation, which is a great advantage and can achieve a satisfactory therapeutic effect (Figs. 1, 2).

The factors that prevent the success of closed reduction are mainly the embedding of tissues in the broken end of the fracture, mainly periosteum, long head tendon of biceps brachii, deltoid muscle, and crushed fracture fragments [22]. In the patients we treat, the main cause of failure to close reduction is the incarceration of the periosteum. Although some studies show that the incarceration of the long head tendon of the biceps brachii at the broken end of the fracture is the main cause of the open reduction, other studies do not support this conclusion [1]. Some researchers found that the biceps tendon is seldom stuck in the broken end of the fracture when the proximal humerus fracture [1]. In our study, incarceration of the biceps brachii tendon during surgery was not found. The main factor impeding fracture reduction is the embedding of the periosteum. We cut the embedded periosteum during surgery to remove obstructions to periosteum reduction. If three closed reductions failed, the open reduction was performed using an anterior shoulder incision [23]. Open reduction is increasingly accepted as an acceptable treatment for adolescent proximal humerus fractures with significant displacement of the fracture end and failure of closed reduction [22]. In our study, satisfactory results have also been obtained in the reduction of proximal humerus fractures in adolescents with failed closure reduction.

Pin tract infection is a major complication in the treatment of pediatric fractures with external fixation. The main reason is that Schanz screws are prone to pin tract infection due to their large diameter. Most pin tract infections are mild and can be managed with dressing changes, and topical or systemic antibiotics [24]. The skin scar after the external fixator operation is also large, which may affect the appearance. Avoiding tension in the skin around the Schanz screw and Kirschner wire reduces the risk of complications such as infection and scarring. Because of the specific anatomy of the shoulder joint, the proximal Schanz screw may be at risk for causing joint infections. Osteonecrosis of the humeral head is an uncommon complication [25] that is not encountered in our clinical work. There is also a need to focus on the problem of pathological fractures. It is necessary to read the film carefully before the operation to rule out pathological fracture, otherwise, serious consequences may occur.

Although there is no standard score for shoulder function after pediatric proximal humeral fracture, adolescents can generally thoroughly understand the questionnaire of various scores and answer it independently [26] In a recent review, the authors favored an individualized surgical design for N–H type 3 and 4 proximal humerus fractures in adolescents. [27]. Fractures in older adolescents take longer to heal, so immobile time is correspondingly longer. Moreover, the bone development of girls is earlier than that of boys, which should also be paid attention to in the choice of surgical treatment [5]. The advantage of our choice of the double-Schanz screw external fixator combined with anti-rotating Kirschner wire surgical treatment was its ability to achieve functional movement of the shoulder joint in the early postoperative period. It did not require prolonged postoperative immobilization of the affected limb, improves the quality of life of patients after surgery, and can shorten the recovery time of adolescents with proximal humeral fractures compared to other surgical methods.

At the same time, it should be noted that there can be differences between the actual age of the child and the physiological age. Many children aged 8–10 years have reached the physique of adolescents aged 12–14 years, so it is necessary to make individualized and careful treatment plans [28].

The drawback of the number of cases studied was small because of the low incidence of pediatric proximal humeral fractures. Also, this study was a retrospective analysis of the efficacy of one surgical method and has not been compared with other surgical methods. We will further improve it in future studies.

## Conclusion

The double-Schanz screw external fixator combined with anti-rotating Kirschner wire is a safe and effective treatment for displaced Salter–Harris type II proximal humerus fractures in skeletally immature patients over the age of 10 years.

## Data Availability

All data generated or analyzed during this study are included in this manuscript.
