# Correction: Efficacy analysis of a double-Schanz screw external fixator combined with anti-rotating Kirschner wire in the treatment of proximal humerus fractures in skeletally immature patients

**DOI:** 10.1186/s13018-023-03722-8

**Published:** 2023-03-23

**Authors:** Qian Wang, Yu Wang, Huai Zhao, Qingzhu Kong, Jingxin Zhao, Yu jin

**Affiliations:** 1 Trauma Department of Orthopedics, Affiliated Hospital of Chengde Medical College, 36 Nanyingzi Street, Shuangqiao District, Chengde, 067000 Hebei People’s Republic of China; 2 Department of General Surgery, Shengjing Hospital Affiliated China Medical University, Shenyang, 110004 Liaoning People’s Republic of China

**Correction: Journal of Orthopaedic Surgery and Research (2022) 17:544** 10.1186/s13018-022-03434-5

Following publication of the original article [1], the authors identified an error in the author name of Jingxin Zhao.

The incorrect author name is: Jingxing Zhao

The correct author name is: Jingxin Zhao

The author group has been updated above and the original article [1] has been corrected.
